# Stroke Prevention and Cognitive Reserve: Emerging Approaches to Modifying Risk and Delaying Onset of Dementia

**DOI:** 10.3389/fneur.2013.00013

**Published:** 2013-03-21

**Authors:** Kevin J. Willis, Antoine M. Hakim

**Affiliations:** ^1^Canadian Stroke NetworkOttawa, ON, Canada; ^2^Neuroscience Research, Ottawa Hospital Research InstituteOttawa, ON, Canada; ^3^Brain and Mind Research Institute, University of OttawaOttawa, ON, Canada

**Keywords:** stroke, Alzheimer’s, prevention, cognitive reserve, dementia

## Abstract

Demographic changes and improvements in health care are projected to result in dramatic increases in the prevalence of dementia. Alzheimer’s disease is widely considered to be the primary cause of dementia – a disease for which there is currently no cure nor effective treatment, and for which it is thought that little can be done to mitigate risk. However, an increasing understanding of the role and extent of vascular contributions to the development of dementia, and appreciation of the interactions between stroke and Alzheimer’s disease, suggest that targeting vascular risk factors may be very beneficial in reducing the impact of dementia. We also describe how active stimulation of the brain throughout the life course builds cognitive reserve that can offset or compensate for cognitive decline in later life. Finally, we discuss the implications of these emerging approaches for dementia prevention and advocate for the urgent implementation of more extensive public health strategies to improve vascular health.

## Introduction

Dementia is a debilitating syndrome of brain disorders distinguished by a progressive decline in cognitive functions to the point where everyday activities are compromised. Demographic changes and improvements in health care are driving dramatic increases in the numbers and proportion of people reaching old age, and advanced age is a powerful risk factor for developing dementia. As a consequence, a staggering growth in the number of people with dementia is projected, reaching 65.7 million globally by 2030 (World Health Organization and Alzheimer’s Disease International, 2012). It is difficult to understate the health and economic burden that this will generate. A range of degenerative and other neuropathological processes can result in dementia, but Alzheimer’s disease is widely considered to be the most frequent cause – although this view is changing, as we will discuss. Alzheimer’s disease is defined by a signature brain pathology characterized by amyloid peptide deposits in blood vessels and in plaques, by the formation of neurofibrillary tangles, and by neurodegeneration. The cause of late-onset Alzheimer’s disease is unknown and attempts to develop pharmacological solutions have largely focused on eliminating the amyloid deposition that is hypothesized to be neurotoxic. However, the causal role of amyloid deposition has been questioned (de la Torre, 2004) and anti-amyloid immunotherapy drugs that clear amyloid fail to impact cognitive or functional outcomes (BBC News, 2012; Teich and Arancio, 2012). Extensive trials of ginkgo biloba extracts that in preclinical studies exhibit neuroprotective and anti-amyloid actions have also failed to show clinical efficacy (Vellas et al., 2012). Regrettably, little can presently be offered to help those who are diagnosed with Alzheimer’s disease (Spence, 2012), no effective treatment is available, future prospects for brain repair through approaches such as functional neurogenesis are increasingly uncertain (Costandi, 2012), and it has been argued that significant cognitive loss is incurable (de la Torre, 2010). Adding to the negative outlook is a report from an expert independent panel recently commissioned by the National Institutes of Health that concluded that insufficient evidence was available to support recommendations for the modification of risk for Alzheimer’s disease or cognitive decline (National Institutes of Health, 2010; Daviglus et al., 2011).

This pessimistic view that dementia is largely an inevitable consequence of aging is challenged by international comparisons that show lower incidence rates in developing compared to developed countries (Hendrie et al., 2001; World Health Organization and Alzheimers Disease International, 2012). There are also indications that within developed countries there have been decreases in dementia incidence over recent decades that are associated with improvements in social and medical factors (Schrijvers et al., 2012). Thus, dementia risk appears to be modifiable.

In this perspective we provide an overview of two areas of active investigation that are revealing potential opportunities to reduce the impact of the dementia epidemic: a reduction in dementia resulting from stroke, and an expansion in cognitive reserve. Firstly, vascular disease and stroke leading to dementia have received less attention than Alzheimer’s disease, and “vascular dementia” is thought of as the second most common cause of dementia. There is however an increasing appreciation that vascular contributions to the development of dementia are significant (Gorelick et al., 2011) and that dementia pathogenesis is typically complex, involving interacting vascular and degenerative processes (Korczyn et al., 2012). This implies that vascular disease prevention may represent an effective approach to decreasing dementia risk and delaying its onset. Secondly, the lack of a direct relationship between the extent of brain damage or pathology and the degree of cognitive decline evident clinically, in combination with epidemiological evidence linking resistance to cognitive decline with extended education, social, and intellectual activity, have led to the cognitive reserve hypothesis (Stern, 2002). Life-long cognitive activity to enhance cognitive reserve may also represent a promising strategy to mitigate cognitive decline and postpone dementia.

## Clinical Stroke and Dementia

Thomas Willis first described that dementia can result from a large stroke in 1672 (Romàn, 2002a) and it is now well known that stroke doubles the risk of new-onset dementia (Leys et al., 2005). An extensive systematic review found that dementia prevalence among persons with clinical stroke was 10% prior to first stroke and that an additional 10% develop new dementia shortly after first stroke (Pendlebury and Rothwell, 2009). The occurrence of a second stroke is a powerful predictor of dementia and among persons with recurrent stroke the prevalence of dementia is 30%. The prevalence of cognitive impairment is even higher: 50% of individuals exhibit deficits when assessed 3 months post-stroke (Sundar and Adwani, 2010). Stroke can therefore initiate rapid cognitive decline. This forms a basis for the etiology of vascular dementia and the more recent, broader concept of vascular cognitive impairment, which encompasses mild cognitive impairment resulting from vascular disease processes through to dementia following stroke (Hachinski et al., 2006).

## Covert Stroke, Microemboli, and Dementia

Infarcts and hemorrhages arising from stroke and other cerebrovascular disease can vary in size, number, and location and may or may not be associated with the clinical signs and symptoms of stroke when they occur. Advances in imaging technology are revealing the widespread extent of subclinical cerebrovascular brain injury in the general senior population, even in individuals with no prior history of stroke. Small (<1 cm) infarcts arising from clinically unrecognized “covert” strokes have an overall prevalence up to fivefold that of clinical stroke, ranging from around 10% at age 50 to almost 30% by age 85 (Vermeer et al., 2007). Cerebral microbleeds – the hemorrhagic counterpart of small ischemic strokes, are also common (Jeerakathil et al., 2004; Poels et al., 2010) with a prevalence of 6% at age 50, rising to 36% among those aged 80 years and older. In addition to age, their frequency is also associated with vascular risk factors (Viswanathan and Chabriat, 2006). Lesions in the white matter are even more common and can also be observed in the brains of most individuals from age 30, the extent, and volume increasing with increasing age (DeCarli et al., 2005). When multiple measures of vascular pathology are included, frequencies of cerebrovascular pathology of 78% have been reported for community-based autopsy series among persons over 70 years (Neuropathology Group. Medical Research Council Cognitive Function and Aging Study, 2001). These covert strokes are not clinically silent as was earlier thought, but are associated with cognitive deficits, particularly executive dysfunction (Werring et al., 2004; Prins et al., 2005), and memory loss (Blum et al., 2012). Covert strokes and white matter lesions each more than double the risk of stroke and the risk of dementia (Vermeer et al., 2003a,b; Debette and Markus, 2010).

Further evidence for a direct role of covert ischemic stroke in the progressive decline of cognitive function is provided by transcranial Doppler studies. Monitoring the middle cerebral artery over the course of several hours can detect spontaneous cerebral microemboli that may arise from carotid disease and atrial fibrillation (Goldberg et al., 2012). Recent Doppler studies detected cerebral microemboli in almost 40% of patients with dementia compared to only 14% in matched controls (Purandare et al., 2006), and over the following 2 years patients with dementia and microemboli exhibited a twofold faster cognitive deterioration when compared to emboli-negative patients with dementia (Purandare et al., 2012). Cerebral microemboli also arise perioperatively during major surgery and this may be the cause of the postoperative cognitive impairment that occurs in over one in four treated patients aged 60 and above (Moller et al., 1998). Congestive heart failure is also a highly prevalent condition among the elderly and cerebral microemboli combined with chronic cerebral hypoperfusion are thought to account for the cognitive decline that commonly accompanies heart failure (Dardiotis et al., 2012).

Based on a consideration of the prevalence and extent of the contributions of clinical and covert stroke to the development of dementia, it has been proposed that vascular dementia may be the most common form of dementia (Romàn, 2002b). However, the heterogeneity of injury arising from stroke and the importance of the lesion site have made it difficult to define both the clinical diagnostic criteria and the pathological criteria that can allow an accurate quantitative evaluation of the vascular contribution to cognitive decline (Grinberg and Heinsen, 2010).

## Stroke and Alzheimer’s Disease

In general clinical practice, Alzheimer’s disease has become synonymous with dementia and “probable Alzheimer’s disease” is the typical diagnosis given to elderly persons with dementia. But autopsy series reveal that around one in four *non*-demented elderly persons have Alzheimer disease pathology (Schneider et al., 2007). On the other hand, among persons who had a clinical diagnosis of probable Alzheimer’s disease or mild cognitive impairment, almost half exhibited mixed Alzheimer and cerebral infarct pathology (Schneider et al., 2009). As visible microscopic infarcts and white matter damage were not included in this analysis, the true proportion of mixed pathologies is likely to be significantly higher. Furthermore, studies that combine prospective neuropsychological examination with a neuropathological assessment at death find that in individuals with post-mortem Alzheimer’s pathology, the presence of deep brain infarcts is associated with a more extensive cognitive decline prior to death (Snowdon et al., 1997). Additionally, these studies have established that macroscopic infarcts independently reduce cognition and increase the likelihood of dementia, and that vascular pathology may also be associated with episodic memory loss – the signature Alzheimer phenotype (Schneider and Bennett, 2010). What emerges is a more complex picture wherein cognitive decline and dementia are typically associated with co-occurring, interacting Alzheimer and vascular disease processes, each with overlapping clinical features.

It is also becoming apparent that vascular disease processes and Alzheimer pathology interact at the level of the neurovascular unit. As described in a recent extensive review of the field (Pimentel-Coelho and Rivest, 2012), cerebral ischemia and hypoxia may both increase the expression and reduce the clearance, of amyloid proteins. Also, the deposition of Alzheimer proteins in neurons and in the walls of small-vessels increases their susceptibility to injury from hypoxia-ischemia. In particular, cerebral amyloid angiopathy, in which amyloid protein accumulates in the walls of small-vessels in the brain, causes microbleeds and may underlie the cerebral hypoperfusion that is associated with the early stages of cognitive decline.

## Vascular Risk and Dementia

In view of the contribution that vascular disease processes (especially stroke) make to the development of dementia, it is not surprising that epidemiological studies find evidence for the association of modifiable vascular risk factors such as mid-life hypertension (Nagai et al., 2010), diabetes (Lu et al., 2009), cholesterol (van Vliet, 2012), poor diet (Solfrizzi et al., 2011), obesity (Lee, 2011), physical inactivity (Denkinger et al., 2012), and smoking (Rusanen et al., 2010), with increased likelihood of Alzheimer’s disease (Patterson et al., 2007; Barnes and Yaffe, 2011). It has been observed however (Chui et al., 2012), that the associations established are most consistent with increased risk of dementia rather than increased risk of Alzheimer’s disease defined by its pathology. This also holds for depression, now accepted to be a driver of vascular compromise and cognitive impairment (Hakim, 2011).

There is also observational data indicating that managing vascular risk factors can slow cognitive decline, even among patients with dementia (Deschaintre et al., 2009). To date randomized controlled trials have largely failed to establish that treatment with medications targeting vascular risk factors such as hypertension, dyslipidemia, and inflammation are effective in dementia prevention. These negative results have been attributed to methodological issues, most notably that the intervention trials were underpowered for dementia, of short duration, and focused on late rather than mid-life disease. A series of ongoing trials have been designed to address these limitations (Mangialasche et al., 2012; Qiu, 2012).

## Cognitive Reserve

It is frequently observed that individuals who are cognitively normal prior to death can still exhibit an extensive burden of neuropathology upon post-mortem examination. Data from studies of large cohorts of elderly people indicate that brain weight and pathology burden only account for one third to half of the observed variance in cognitive ability (Dowling et al., 2011). The cognitive reserve hypothesis explains this disconnect in terms of brain resilience due to factors such as capacity to withstand damage, ability to compensate for damage through the use of alternate networks, and remodeling and plasticity (Stern, 2006; Esiri and Chance, 2012). Engaging in stimulating mental and physical activity throughout life is thought to increase cognitive reserve which in turn slows cognitive decline and delays onset of dementia. There is strong observational evidence that high versus low levels of education protects against dementia, approximately halving risk (Meng and D’Arcy, 2012; Prince et al., 2012). Education also protects cognitive function in inherited early onset small vessel disease – i.e., “pure” vascular cognitive impairment (Zieren et al., 2013). In addition, educational history is an independent predictor of post-stroke cognitive deficits (Ojala-Oksala et al., 2012). Participation in social networks may also be beneficial (Bennett et al., 2006). Compelling support for the cognitive reserve hypothesis is provided by the advantageous effect of bilingualism, but only if the additional language is actively used. Diagnosis of dementia is found to be delayed by 3–4 years when otherwise equivalent mono and bilingual dementia cases are compared (Bialystok et al., 2007, 2012). If the onset of dementia among the global population could be delayed by just 2 years it has been estimated that almost a quarter of the cases of Alzheimer’s disease expected by the year 2050 could be avoided (Brookmeyer et al., 2007). Thus the concept of cognitive reserve is worthy of exploration and further study.

Cognitive training programs are actively marketed to older adults as a way to offset age-associated cognitive decline. Results from interventional trials indicate that cognitive training can improve specific cognitive ability, but that the benefits are mostly limited to the targeted outcome (Ball et al., 2002; Acevedo and Loewenstein, 2007; Owen et al., 2010). Studies of cognitive training and other related cognitive interventions in the elderly population conducted to date have lacked the power and have been too diverse in approach to establish whether such programs are effective at delaying or preventing dementia onset (Buschert et al., 2010).

## Public Health and Other Implications

Alzheimer’s disease has become enshrined in our popular culture and is commonly thought by the public and health professionals to be exclusively responsible for cognitive decline and the projected epidemic of elderly persons with dementia. In contrast, the typical mixed pathology nature of late-onset dementia and the vascular contribution to cognitive decline are largely unrecognized. Furthermore, the view prevails that the onset of “Alzheimer’s” is a mostly unavoidable consequence of reaching old age. This review therefore has a number of implications.

Dementia prevention should be considered from a broader perspective than just “pure” Alzheimer’s disease. The potential that vascular disease prevention offers for dementia represents a major overlooked opportunity. Early (by mid-life) prevention and treatment of the well established and highly prevalent vascular risk factors such as hypertension and diabetes, in combination with lifestyle changes that increase physical activity, improve diet, and eliminate smoking are likely to significantly reduce the incidence of dementia at the population level (Ahlskog et al., 2011; Willis et al., 2012). Since recurrent events accelerate cognitive decline, improvements in the secondary prevention of stroke and heart disease are also likely to positively impact dementia rates. There is currently no level I evidence from randomized controlled trials that support the role of vascular risk factors in dementia prevention. Such evidence may not be feasible to obtain, as conducting randomized controlled trials over many decades presents significant challenges, and it may not be possible for ethical reasons to investigate certain vascular risk factors in intervention trials (Kroke et al., 2003). However, the level II evidence that we have outlined provides a strong argument for an urgent enhancement of public health measures to improve vascular health as part of a population level dementia prevention strategy. Importantly, it adds further justification for population level vascular health interventions such as smoking legislation, banning trans fat, and decreasing sodium levels in the food supply.

In addition to the gap in translating the current knowledge of the role of vascular disease and stroke into dementia prevention, there is also a significant gap in awareness among health professionals and the public. Physician training is necessary to develop their ability to act as advocates and educators on healthy brain aging (Romàn et al., 2012). Furthermore, governments, cardiovascular and Alzheimer’s disease organizations, and charities should work together to ensure that this message reaches the general public.

We need to break down the barriers that result in research into the disease processes that cause dementia taking separate and largely non-communicating paths. New research networks should be formed that bring scientists from vascular, stroke, and amyloid research, together with clinicians from the fields of neurology, psychiatry, and gerontology. Such networks would establish collaborations to study the prevention strategies and treatments, as well as repair modalities, that may be effective in alleviating cognitive impairment and dementia.

A modest delay in the onset of dementia, such as what could potentially be achievable through enhancing cognitive reserve, can significantly reduce incidence of the disease. Public policy should continue to promote universal education and also support the maintenance of lifestyles that maximize physical, mental, and social activity into advanced age. While cognitive training in late life may not add appreciably to the reserve built over a lifetime, it is clear that the social isolation and unstimulating environments experienced by many elderly will negatively impact their brain health.

The disappointing results from candidate drugs targeting Alzheimer pathology and our emerging understanding of the vascular contributions to cognitive decline make it increasingly unlikely that we will be able to change the course of established disease for the foreseeable future. However, there is much to be gained by increasing efforts targeting diet, physical activity, vascular interventions, and enhancing cognitive reserve (Table 1). The time to act is now.

**Table 1:** **Interventions to reduce dementia risk**.

Lifestyle*	Diet*	Psychosocial	Pharmacotherapy*
Smoking cessation	DASH-style diet^c^	Extensive education	Hypertension
Moderation of alcohol intake^a^	Rich in vegetables and fruit	Cognitive engagement	Atrial fibrillation
Weight control	Reduced intake of sodium	Social stimulation	Recurrent stroke
Physical activity^b^	Free of trans fat	Avoidance/treatment of depression	Diabetes and hyperlipidemia

*Based on recommendations for the prevention of stroke (Goldstein et al., 2011).

*^a^≤2 Drinks per day for men and ≤1 drink per day for women*.

*^b^150 min per week of moderate intensity or 75 min per week of vigorous intensity aerobic physical activity*.

*^c^Emphasizes consumption of fruits, vegetables, and low-fat dairy products and is reduced in saturated fat*.

## Conflict of Interest Statement

The authors declare that the research was conducted in the absence of any commercial or financial relationships that could be construed as a potential conflict of interest.
